# Supplementary material for: CellSighter: a neural network to classify cells in highly multiplexed images
Source: Nat Commun. 2023 Jul 18;14:4302. doi: 10.1038/s41467-023-40066-7 (PMC10354029; doi:10.1038/s41467-023-40066-7)
Supplement: Supplementary file 1 — Supplementary Information [file 41467_2023_40066_MOESM1_ESM.pdf]

Supplementary information

CellSighter: A neural network to classify cells in highly multiplexed images

Yael Amitay, Yuval Bussi, Ben Feinstein, Shai Bagon, Idan Milo, Leeat Keren

Figure S1

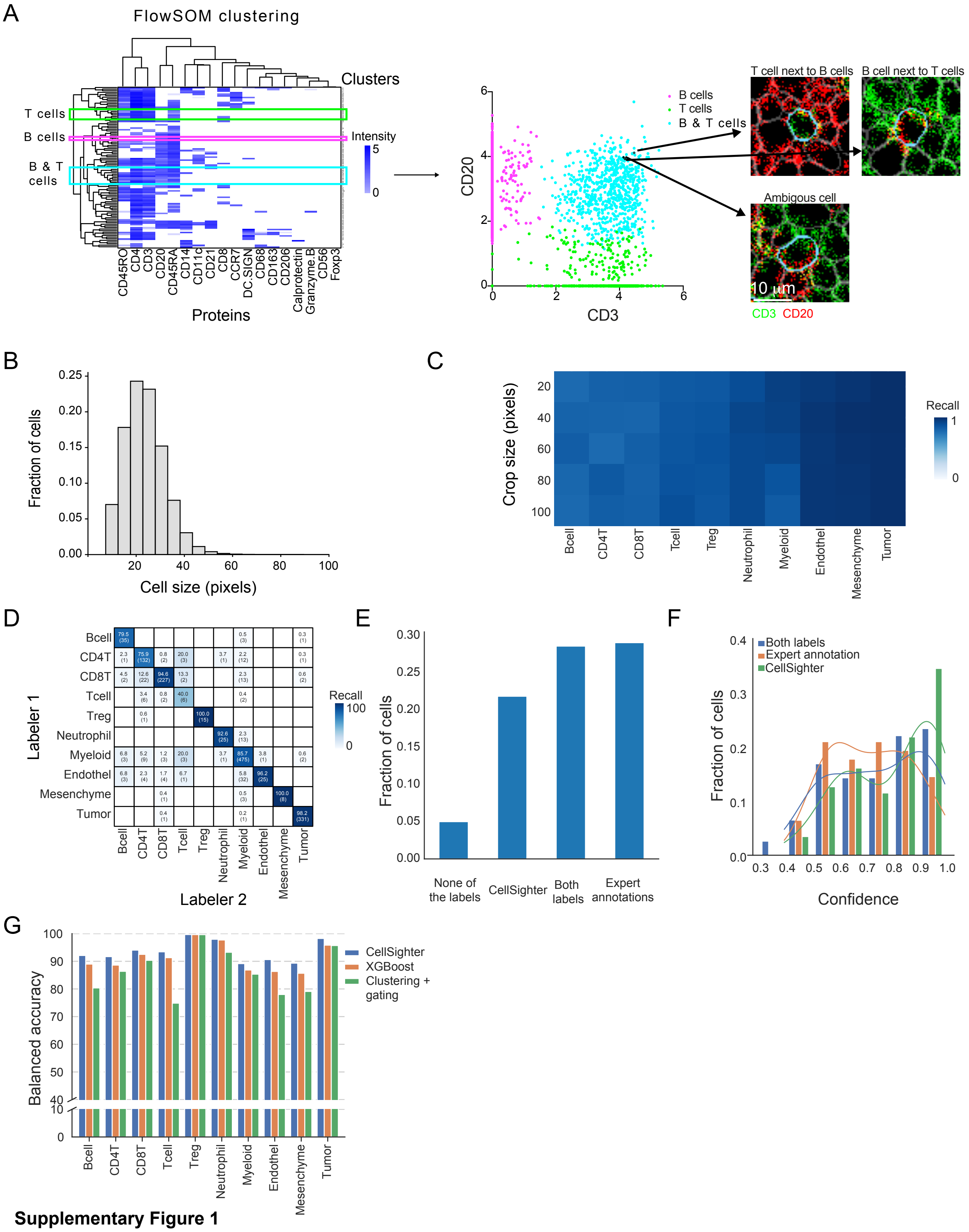

Supplementary Figure 1

(A) Clustering expression vectors yields ambiguous cell classes. The expression vectors for all cells in the dataset were clustered to 100 clusters using FlowSOM (left). Each cluster was then manually annotated. The right panel shows a scatter plot of the cells from the clusters marked by the green, pink and blue boxes. For each cell, shown is its expression of CD3 (x-axis) and CD20 (y-axis). Cells from the blue cluster are difficult to classify, and encompass T cells next to B cells, B cells next to T cells and ambiguous cells, which could plausibly be either T cells or B cells. **(B)** Histogram showing the major axis length in pixels for all cells in the Melanoma dataset. **(C)** Shown is the agreement between CellSighter and expert labeling (recall, blue) for different cell classes (x-axis) when varying the crop size of the input into CellSighter between 20 and 100 pixels. **(D)** For the melanoma dataset, shown is the agreement on the labels of all cells from a single FOV between two different human labelers. **(E)** For the additional cells differing in classification between CellSighter and expert annotation we assessed what fraction of the expert annotations were in CellSighter's top two classification predictions (above probability 0.3). In cases in which inspection suggested that the expert was correct (Expert annotation and both) CellSighter had a higher chance of including this label in its top two classifications (29% and 28% respectively), whereas in cases in which inspection suggested that the expert was incorrect (CellSighter and None) CellSighter had a lower chance of including this label in the top two (22% and 5% respectively). **(F)** For the additional cells differing in classification between CellSighter and expert annotation, shown is CellSighter's confidence in prediction for different classes as determined by expert inspection. We find that in cases in which CellSighter is correct (blue), it has higher confidence than in cases where it was erroneous (orange). **(G)** Comparison of the balanced accuracy (y-axis) for each cell class (x-axis) of CellSighter (blue), XGBoost (orange) and Clustering and gating (green).

Figure S2

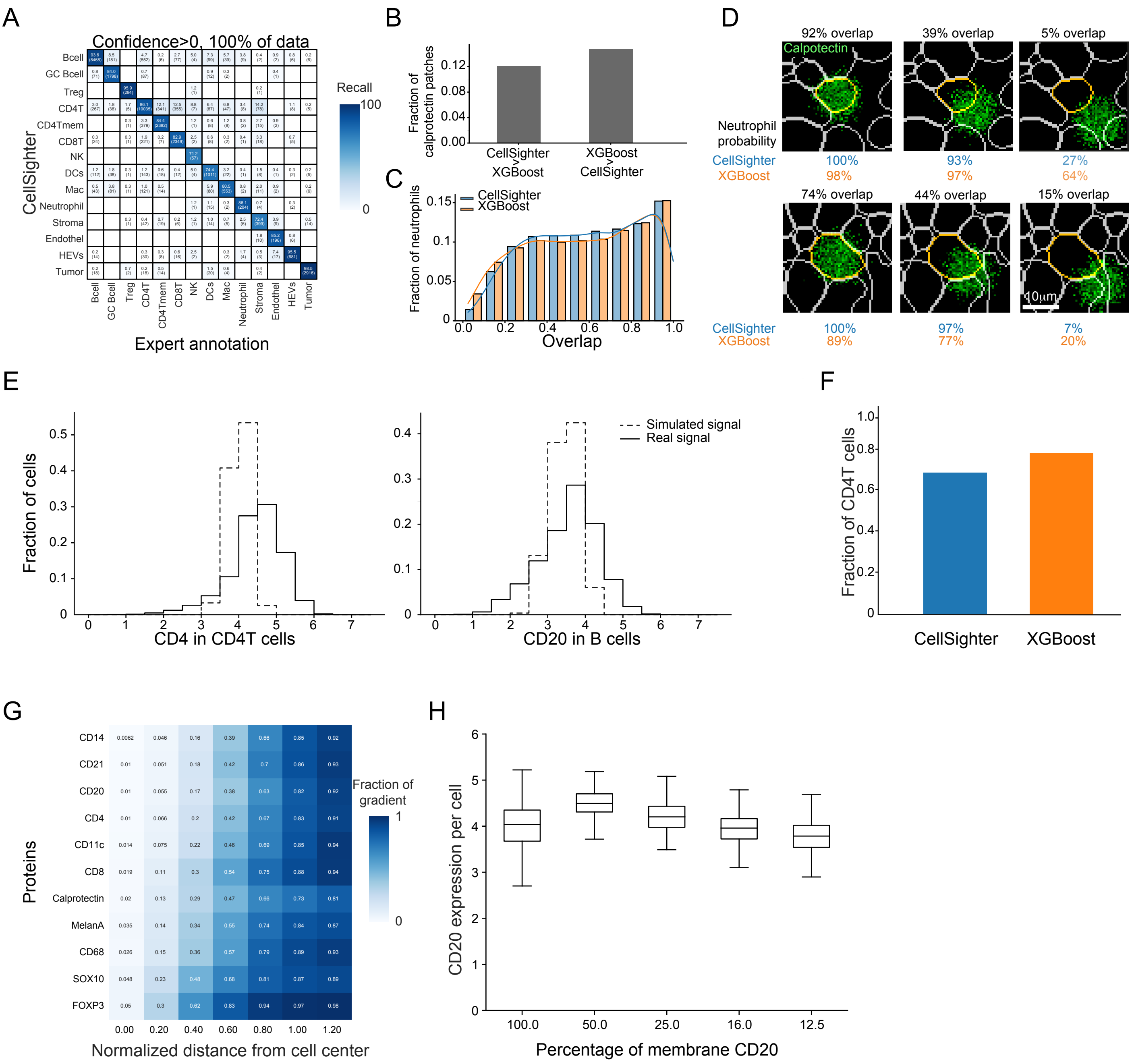

Supplementary Figure 2

(A) Comparison between labels generated by experts using clustering, gating and rounds of manual annotation (x-axis) and labels generated by CellSighter (y-axis) for the melanoma lymph node metastases dataset.**(B)** The number of cells classified as Neutrophils by XGBoost or CellSighter was quantified for all patches of Calprotectin signal. Bar graphs show the proportion of calprotectin signal patches (y-axis) in which the number of Neutrophils predicted was larger in either CellSighter (left) or XGBoost (right) respectively. **(C)** Results of simulations for 237 cells with different overlaps of Calprotectin as shown in (D). Shown are the proportion of cells classified as Neutrophils (y-axis) at different levels of intersection of their Calprotectin signal with the cell segmentation (x-axis). At low intersections, CellSighter (blue) classifies less cells as Neutrophils than XGBoost (orange). **(D)** Example images from simulations in which the level of intersection of Calprotectin (green) with a cell-to-be-classified (yellow) is varied. Shown are three degrees of overlap for two cells. **(E)** Histogram showing expression levels of CD4 in CD4 T cells (left) or CD20 in B cells (right) for real (black) and simulated (dashed) data. **(F)** Crops centered on CD4 T cells had their cognate CD4 and CD20 signals removed and CD4 was reintroduced as a membranous signal. Shown are results for the fraction of these cells that were classified as CD4 T cells (y-axis) by CellSighter and XGBoost (x-axis). **(G)** Shown is the normalized sum of gradients (y-axis) as a function of the normalized integrated distance from the cell center (x-axis) for different lineage proteins. The gradients for each protein were evaluated in its respective cell type: CD14 – Macrophages, CD21 – GC B cells, CD20 – B cells, CD4 – T helper cells, CD11c – DCs, CD8 – CD8 T cells, Calprotectin – Neutrophils, MelanA – Tumor cells, CD68 – Macrophages, SOX10 – Tumor cells, FOXP3 – Tregs. **(H)** For 200 cells in which CD20 was simulated as a membranous signal with varying overlap with the membrane, shown is the CD20 expression per cell (y-axis) as a function of the percentage of membrane that has CD20 signal (x-axis). Boxplots show median, first and the third quartile. Whiskers reach up to $1.5\cdot(Q_3 - Q_1)$ from the end of the box. Dots denote outliers.

Figure S3

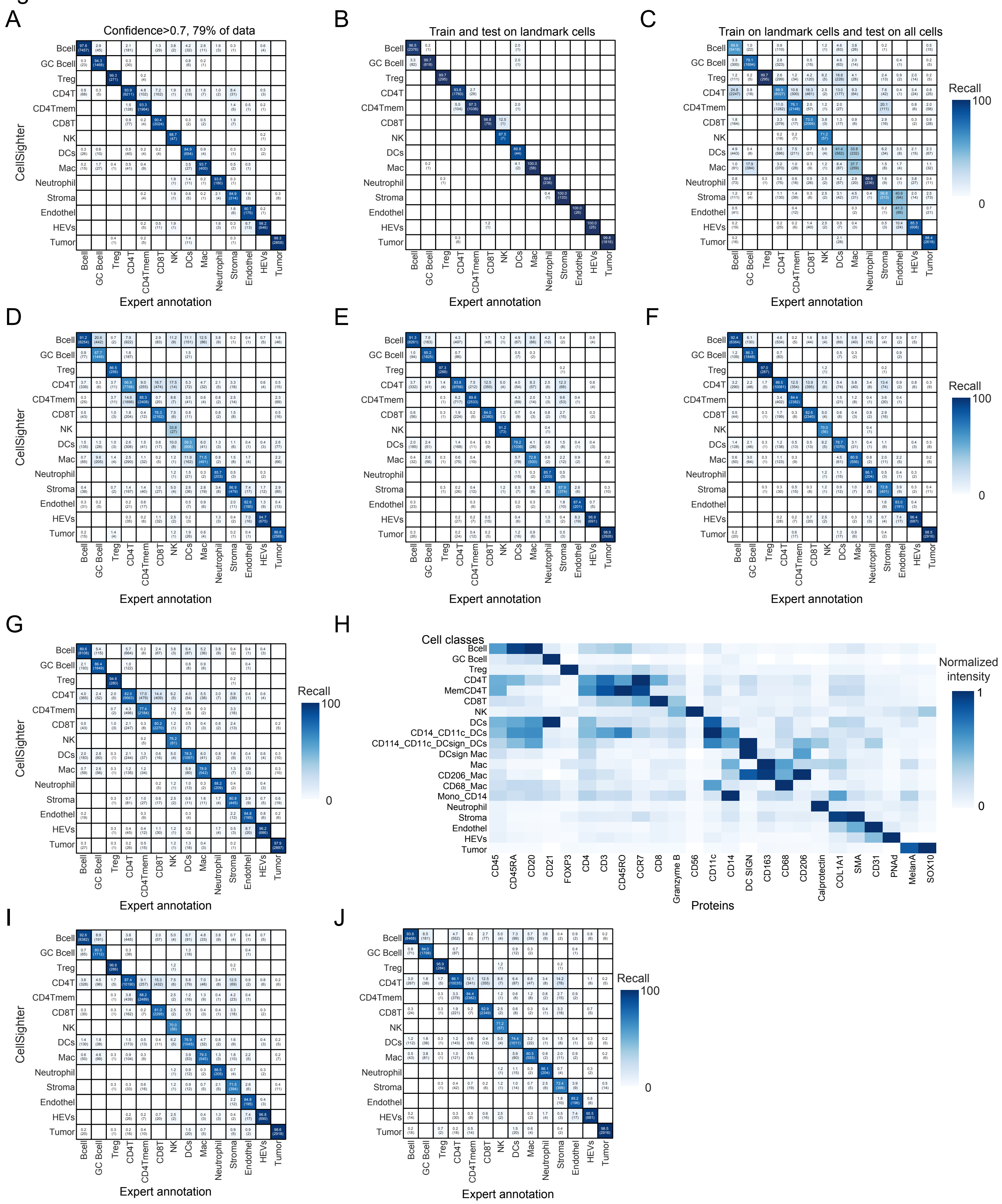

Supplementary Figure 3

(A-G,I,J) Comparison between labels generated by experts using clustering, gating and manual annotation (x-axis) and labels generated by CellSighter (y-axis). Each panel shows the results of different training schemes. **(A)** Baseline model was run, as in supp. Figure 2A. Shown are the results for high-confidence classifications (Confidence>0.7). **(B)** Shown are results for a model that was trained and evaluated on landmark cells. **(C)** Shown are results for a model that was trained on landmark cells and evaluated on the complete dataset. **(D)** Shown are results for a model that was trained after applying Anscombe transformation on the images. **(E)** Same as (D), using 99th percentile normalization on the images. **(F)** Shown are results for a model that was trained on 39 proteins, including both lineage and functional proteins. **(G)** Shown are results for a model that was trained after applying a 2x2 kernel to artificially reduce the resolution to 1mm/pixel **(H)** For the melanoma lymph node metastases data, shown is the expression of distinct proteins (x-axis) across cell classes (y-axis) normalized by rows and columns. Macrophages and DCs were over-clustered to finer-grained subtypes. **(I)** Similar to Fig S2 A, CellSighter was trained on “DCs” and “Macrophages”. **(J)** Similar to Fig S2 A, CellSighter was trained on over-clustered subsets of "DCs" and "Macrophages". In the test set, the over-clustered subsets were merged to "DCs" and "Macrophages".

Figure S4

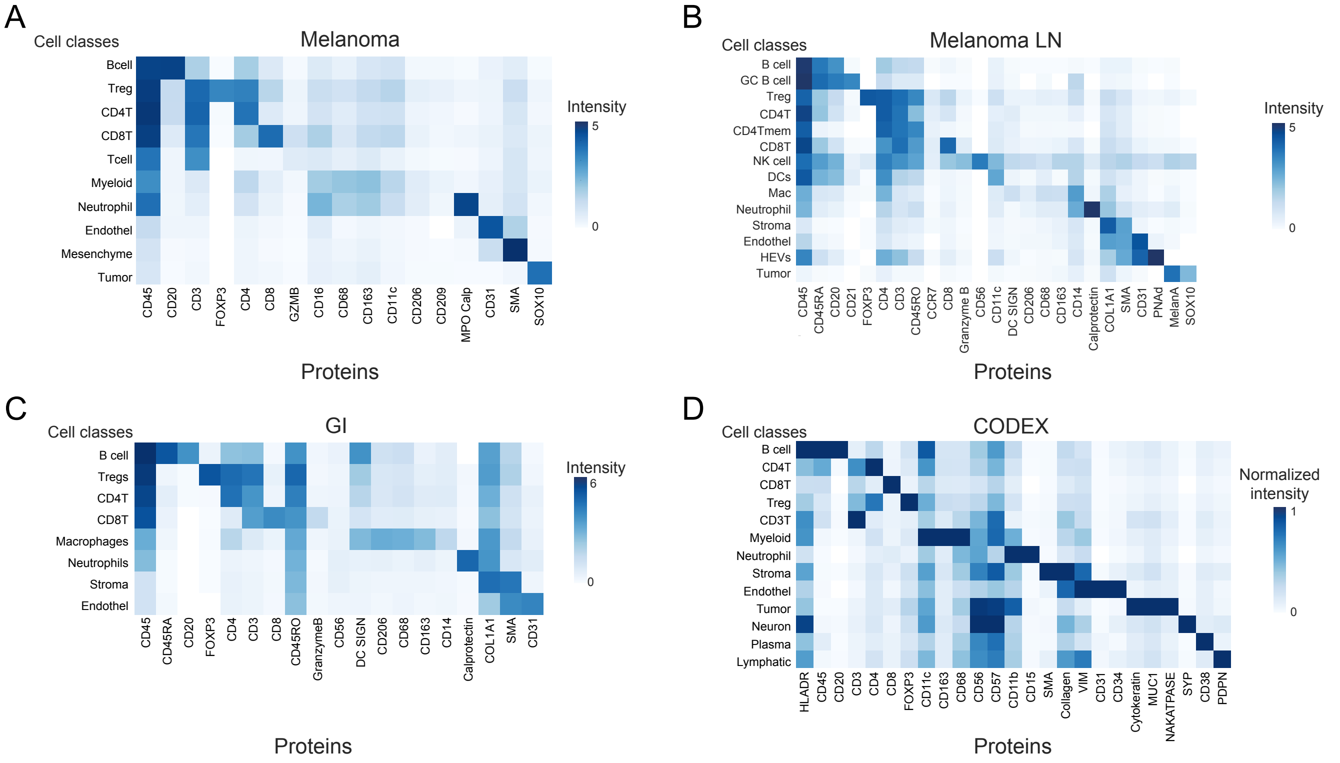

Supplementary Figure 4
(A) For the melanoma data, shown is expression of distinct proteins (x-axis) across cell classes (y-axis). **(B)** For the melanoma lymph node metastases data, shown is expression of distinct proteins (x-axis) across cell classes (y-axis). **(C)** For the gastrointestinal data, shown is expression of distinct proteins (x-axis) across cell classes (y-axis). **(D)** For the CODEX data, shown is expression of distinct proteins (x-axis) across cell classes (y-axis) values were exponential transformed and normalized by rows and columns for visualization.
